# Theoretical fractional formulation of a three-dimensional radio frequency ion trap (Paul-trap) for optimum mass separation

**DOI:** 10.1177/14690667211026790

**Published:** 2021-07-04

**Authors:** Sarkhosh Seddighi Chaharborj, Shahriar Seddighi Chaharborj, Zahra Seddighi Chaharborj, Pei See Phang

**Affiliations:** 1 School of Mathematics and Statistics, Carleton University, Ottawa, ON, Canada; 2 Department of Mathematical Sciences, Faculty of Science, Universiti Teknologi Malaysia, Johor Bahru, Malaysia; 3 Department of Mathematics, Faculty of Science, Tabriz Branch, Islamic Azad University, Tabriz, Iran; 4 Faculty of Information Technology and Computer Engineering, Azarbaijan Shahid Madani University, Tabriz, Iran; 5 Department of Mathematics, Faculty of Science, Universiti Putra Malaysia, Seri Kembangan, Malaysia; 6 Brilliant Student Care, Singapore

**Keywords:** Paul–trap, cantor-type, cylindrical coordinate, fractional parameter, fraction ion motion, mass separation

## Abstract

We investigate the dynamics of an ion confined in a Paul–trap supplied by a fractional periodic impulsional potential. The Cantor–type cylindrical coordinate method is a powerful tool to convert differential equations on Cantor sets from cantorian–coordinate systems to Cantor–type cylindrical coordinate systems. By applying this method to the classical Laplace equation, a fractional Laplace equation in the Cantor–type cylindrical coordinate is obtained. The fractional Laplace equation is solved in the Cantor–type cylindrical coordinate, then the ions is modelled and studied for confined ions inside a Paul–trap characterized by a fractional potential. In addition, the effect of the fractional parameter on the stability regions, ion trajectories, phase space, maximum trapping voltage, spacing between two signals and fractional resolution is investigated and discussed.

## Introduction

A brief summary of ion traps and fractional calculus techniques are presented here.

### Ion traps

Quadrupole ion traps were invented at the beginning of the 1950s^1,2,3^ by Paul et al., demonstrating to be excellent tools to perform mass spectrometry.^4–10^Other applications of quadrupole ion traps include quantum computing, ultraprecise atomic clocks, ion crystals, high–precision spectroscopy, fractional ion traps, and etc.^3–12^ Moreover, the combined (Paul and Penning) trap^13,14^ or the Kingdon trap^15^ can be successfully used to achieve mass spectrometry with very good results. Hu et al.^18^ proposed the Orbitrap that can be used as a multi-purpose mass spectrometer to examine different types of chemical systems. High resolution, high-mass accuracy and high dynamic range are interesting features of the Orbitrap.^18–20^

The cylindrical geometry Paul–trap is easier to design and machine with respect to the hyperbolic geometry trap, and that is why it is increasingly attracting interests.^21,22^ Experiments show that the cylindrical ion trap has a good resolution so as to perform mass separation of ions. In addition, its relatively simple geometry and small dimensions make it very suited for ion trapping experiments. Although it is possible to confine particles with distinct charge-to-mass ratios in a Paul trap, this occurs for weakly confined species that are expelled apart from the trap center. Akerman et al. studied the nonlinear mechanical response of a single laser-cooled ion confined in a linear RF–Paul trap,^23^ demonstrating that both linear and the nonlinear damping components can be completely and accurately controlled. Mihalcea and Vişan^24^ investigate the dynamics of an ion confined in a nonlinear Paul trap, which is shown to behave like a damped parametric oscillator that exhibits fractal properties and complex chaotic orbits.

In this paper, we studied about dynamics of a confined ion in a Paul–trap supplied by a fractional periodic potential. In this regard, the upcoming section studies the fractional Laplace equation in cantor-type cylindrical coordinate. In the next section, the fractional Laplace equation in the cantor-type cylindrical coordinate is modelled and studied. In a further section, ion motion inside a Paul-trap with fractional potential in the cantor-type cylindrical coordinates is modelled. The dynamical system consisting of an ion confined in a Paul–trap is investigated in the penultimate section, where numerical simulations are also performed. The effects of the fractional parameter on the stability regions, ion trajectories, phase space, maximum trapping voltage, spacing between two signals and fractional resolution are reviewed and discussed. In the final section, the results are analyzed and discussed.

### History of fractional calculus

By looking at articles published in recent decades in the fields of science and engineering, we get acquainted with the topics of fractional calculus, differential equations with fractional derivatives, and concepts of this kind. So far, many books and papers in this field have been written from theoretical and practical points of view.^25–31^ The subject of fractional calculus is more than 300 years old. The idea of fractional calculus dates back to the time of basic or classical calculus, and most theories about it were developed before the twentieth century. This was first introduced by Leibniz and L’Hospital’s in 1653.

In the twentieth century, many efforts were made by various scientists in this field. Caputo, by rewriting Riemann–Liouville formula, introduced a new derivative that is now used under the name Caputo derivative. Notable people who have worked on this topic during this period are: Hardy, Samko, Weyl, Riesz and Blair. Since 1970 until now, many people have studied in this field and also left useful articles and books. In this regard, Spanier, Oldham, Miller, Kilbas, Ross and Podlubny can be mentioned. The best resources for studying fractional calculations are books and articles of Miller and Ross, Kilbas and Podlubny. See Ross^32^ for a more comprehensive study of the history of fractional calculus.

### Basic definitions and theorems of the fractional derivatives

Definitions of the fractional derivative of order α>0 are presented in literature.^31,33–40^ The Riemann-Liouville and Caputo fractional derivatives are the most used definitions in our paper.

**Definition 1.1**. *For some*$\alpha \in {\mathbb{R}}^{+}$*, let n be the nearest integer greater than α. The Caputo fractional derivative of order α of a function*h(ξ)*is given by*,^31^

(1) $$D_{*}^{\alpha }h(\xi )=J^{n-\alpha }\frac{d^{n}}{d\xi^{n}}h(\xi )=\frac{1}{\Gamma (n-\alpha )}\int_{0}^{\xi }{\left(\xi -u\right)}^{n-\alpha -1}h^{\left(n\right)}(u)du$$

*with*n−1<α≤n,n∈ℕ.

**Theorem 1.2.***The Riemann-Liouville derivative of order*α>0*with*n−1<α≤n*of the power function*$f(\xi )=\xi^{\beta }$*for*β>0*is given by*,^31^

(2) $$D^{\alpha }\xi^{\beta }=\frac{\Gamma (\beta +1)}{\Gamma (\beta -\alpha +1)}\xi^{\beta -\alpha }$$

**Proof.** Let $h(\xi )=\xi^{\beta }$ (β>0) then we have,

$\frac{d}{d\xi }(\xi^{\beta })=\beta \xi^{\beta -1}\;\;\;\Rightarrow \;\;\;\frac{d^{\alpha }}{d\xi^{\alpha }}\xi^{\beta }=\frac{\beta !}{(\beta -\alpha )!}\xi^{\beta -\alpha },$ replacing the factorials with the “gamma” function leads to,

(3) $$D^{\alpha }\xi^{\beta }=\frac{d^{\alpha }}{d\xi^{\alpha }}\xi^{\beta }=\frac{\Gamma (\beta +1)}{\Gamma (\beta -\alpha +1)}\xi^{\beta -\alpha }$$

**Theorem 1.3.** *The Caputo derivative of order* α>0 *with* n−1<α≤n *of the power function* $f(\xi )=\xi^{\beta }$ *for* β>0 *satisfies*,^31^

(4) $$D_{*}^{\alpha }\xi^{\beta }=\{\begin{array}{l} \frac{\Gamma (\beta +1)}{\Gamma (\beta -\alpha +1)}\xi^{\beta -\alpha };\;\;\;\;\beta >n-1\\ 0;\;\;\;\;\;\;\;\;\;\;\;\;\;\;\;\;\;\;\;\beta \leq n-1 \end{array}$$

**Proof.** *(see proof of Theorem (1.2)).*

## Fractional Laplace equation in the Cantor-type cylindrical coordinate

This section presents the fractional Laplace equation in the Cantor–type cylindrical coordinates. The Cantor–type cylindrical–coordinate method is a powerful tool to convert differential equations on cantor sets from cantorian–coordinate systems to Cantor–type cylindrical coordinate systems.

The cantorian–coordinate system was first described by Yang in 2010.^41,42^ Both fractional and classical differential equations in the coordinate system to cartesian, cylindrical and spherical coordinates are convertible.^43,44^ Newly, the cantorian–coordinate system is set on the fractals problems to obtain acceptable and accurate results. We consider the cantor–type cylindrical coordinates defined in references^42^,^45^ as, $x^{\alpha }=r^{\alpha }\cos_{\alpha }\theta^{\alpha },\;y^{\alpha }=r^{\alpha }\sin_{\alpha }\theta^{\alpha }$ and $z^{\alpha }=z^{\alpha }$, where r∈(0,+∞), z∈(−∞,+∞), θ∈(0,2π) and $r^{2\alpha }=x^{2\alpha }+x^{2\alpha }$. The fractional $\sin_{\alpha }\theta^{\alpha }$ and $\cos_{\alpha }\theta^{\alpha }$ can be defined as follows,

(5) $$\begin{array}{l} \sin_{\alpha }\theta^{\alpha }=\sum_{n=0}^{\infty }{\left(-1\right)}^{n}\frac{\theta^{(2n+1)\alpha }}{\Gamma \left[1+(2n+1)\alpha \right]},\\ \cos_{\alpha }\theta^{\alpha }=\sum_{n=0}^{\infty }{\left(-1\right)}^{n}\frac{\theta^{2\alpha n}}{\Gamma \left[1+2\alpha n\right]} \end{array}$$

Now, according to proposed equations and reference,^45^ we can define the fractional gradient and fractional Laplace operators in the Cantor–type cylindrical coordinate system as follows,

(6) $$\nabla^{\alpha }\Psi (r,\theta ,z)=e_{r}^{\alpha }\frac{\partial^{\alpha }\Psi }{\partial r^{\alpha }}+e_{\theta }^{\alpha }\frac{1}{r^{\alpha }}\frac{\partial^{\alpha }\Psi }{\partial \theta^{\alpha }}+e_{z}^{\alpha }\frac{\partial^{\alpha }\Psi }{\partial z^{\alpha }}$$

(7) $$\nabla^{2\alpha }\Psi (r,\theta ,z)=\frac{\partial^{2\alpha }\Psi }{\partial r^{2\alpha }}+\frac{1}{r^{2\alpha }}\frac{\partial^{2\alpha }\Psi }{\partial \theta^{2\alpha }}+\frac{1}{r^{\alpha }}\frac{\partial^{\alpha }\Psi }{\partial r^{\alpha }}+\frac{\partial^{2\alpha }\Psi }{\partial z^{2\alpha }}$$

where, $e_{r}^{\alpha }=\cos_{\alpha }\theta^{\alpha }e_{1}^{\alpha }+\sin_{\alpha }\theta^{\alpha }e_{2}^{\alpha },\;e_{\theta }^{\alpha }=-\sin_{\alpha }\theta^{\alpha }e_{1}^{\alpha }+\cos_{\alpha }\theta^{\alpha }e_{2}^{\alpha }$ and $e_{z}^{\alpha }=e_{3}^{\alpha }$. Suggested fractional vector was given by, $R=r^{\alpha }\cos_{\alpha }\theta^{\alpha }e_{1}^{\alpha }+r^{\alpha }\sin_{\alpha }\theta^{\alpha }e_{2}^{\alpha }+z^{\alpha }e_{3}^{\alpha }=R_{r}e_{R}^{\alpha }+R_{\theta }e_{\theta }^{\alpha }+R_{z}e_{z}^{\alpha }$.

## Fractional Laplace equation in the Cantor-type cylindrical coordinate

**Figure 1.**
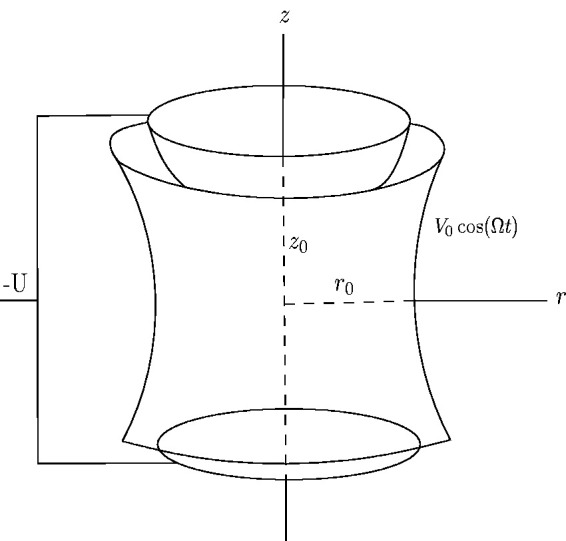
Schematic view of a r.f. Paul–trap.

This section focuses on the fractional Laplace equation in the Cantor–type cylindrical coordinates. The classical 3D Paul trap has a hyperbolic geometry, consisting of a ring and two end cap electrodes that present axial symmetry. In Figure 1, *z*_0_ denotes the distance from the center of the Paul–trap to either of the endcap electrodes, while *r*_0_ denotes the distance from the center of the Paul-trap to the nearest ring surface. Almost any geometry of trap electrodes with ac voltages applied between them, generating a saddle point in the potential, will cater a pseudo–potential minimum in which charged particles can be trapped.^20^ All commonly used mass analyzers use electric and magnetic fields to apply force on charged particles.^1,13^ This force causes the oscillating particle to move around the equilibrium point due to a fractional parabolic potential as, $\Phi^{\alpha }(x,y,z)=A(\gamma_{1}x^{2\alpha }+\gamma_{2}y^{2\alpha }+\gamma_{3}z^{2\alpha })$. Any potential in free space should satisfy the fractional Laplace equation as,

(8) $${▽}^{2\alpha }\Phi^{\alpha }=\frac{\partial^{2\alpha }}{\partial x^{2\alpha }}\Phi^{\alpha }+\frac{\partial^{2\alpha }}{\partial y^{2\alpha }}\Phi^{\alpha }+\frac{\partial^{2\alpha }}{\partial z^{2\alpha }}\Phi^{\alpha }=0$$

where $\frac{\partial^{2\alpha }}{\partial x^{2\alpha }}\Phi^{\alpha },\;\frac{\partial^{2\alpha }}{\partial y^{2\alpha }}\Phi^{\alpha }$ and $\frac{\partial^{2\alpha }}{\partial z^{2\alpha }}\Phi^{\alpha }$ can be computed using the definitions of the fractional derivatives. According to the Theorem (1.2), when α→2α we have,

(9) $$\begin{array}{l} D^{2\alpha }\xi^{\beta }=\frac{\Gamma (\beta +1)}{\Gamma (\beta -2\alpha +1)}\xi^{\beta -2\alpha };\\ \;\;\frac{n-1}{2}<\alpha \leq \frac{n}{2},\;\beta >0 \end{array}$$

let $\xi^{\beta }=x^{2\alpha }$ (2α>0),

(10) $$\begin{array}{l} D^{2\alpha }x^{2\alpha }=\frac{\Gamma (2\alpha +1)}{\Gamma (2\alpha -2\alpha +1)}x^{2\alpha -2\alpha }=\frac{\Gamma (2\alpha +1)}{\Gamma (1)};\\ \;\;\frac{n-1}{2}<\alpha \leq \frac{n}{2},\;2\alpha >0 \end{array}$$

therefore, in the following we have,

(11) $$\begin{array}{l} \frac{\partial^{2\alpha }\Phi^{\alpha }}{\partial x^{2\alpha }}=\frac{A\gamma_{1}\Gamma (2\alpha +1)}{\Gamma (1)},\\ \;\frac{\partial^{2\alpha }\Phi^{\alpha }}{\partial y^{2\alpha }}=\frac{A\gamma_{2}\Gamma (2\alpha +1)}{\Gamma (1)},\\ \frac{\partial^{2\alpha }\Phi^{\alpha }}{\partial z^{2\alpha }}=\frac{A\gamma_{3}\Gamma (2\alpha +1)}{\Gamma (1)} \end{array}$$

from which we obtain,

(12) $${▽}^{2\alpha }\Phi^{\alpha }=\frac{\partial^{2\alpha }\Phi^{\alpha }}{\partial x^{2\alpha }}+\frac{\partial^{2\alpha }\Phi^{\alpha }}{\partial y^{2\alpha }}+\frac{\partial^{2\alpha }\Phi^{\alpha }}{\partial z^{2\alpha }}=A\frac{\Gamma (2\alpha +1)}{\Gamma (1)}[\gamma_{1}+\gamma_{2}+\gamma_{3}]$$

Equation (13) shows that $\gamma_{1}+\gamma_{2}+\gamma_{3}=0$ when ${▽}^{2\alpha }\Phi^{\alpha }=0$. For an ion trap, $\gamma_{1}=\gamma_{2}=1$ and $\gamma_{3}=-2$ and for a quadrupole mass filter $\gamma_{1}=-\gamma_{2}=1$ and $\gamma_{3}=0$. In this paper we focused on the Paul–ion trap, then we assumed, $\gamma_{1}=\gamma_{2}=1$ and $\gamma_{3}=-2$. Therefore, the fractional potential given as, $\Phi^{\alpha }(x,y,z)=A(x^{2\alpha }+y^{2\alpha }-2z^{2\alpha })$. Using the standard transformations $x^{\alpha }=r^{\alpha }\cos_{\alpha }\theta^{\alpha },\;y^{\alpha }=r^{\alpha }\sin_{\alpha }\theta^{\alpha }$ and $z^{\alpha }=z^{\alpha }$, this equation can be transformed into the Cantor-type cylindrical coordinates. Hence, we can derive the fractional potential in the Cantor-type cylindrical coordinates as, $\Phi^{\alpha }(r,z)=A(r^{2\alpha }-2z^{2\alpha })$, with $r^{2\alpha }=r^{2\alpha }\cos_{\alpha }^{2}\theta^{\alpha }+r^{2\alpha }\sin_{\alpha }^{2}\theta^{\alpha }=x^{2\alpha }+y^{2\alpha }$.

This potential can be produced by four hyperbolic electrodes. To obtain this form of electrodes, we can consider the surfaces with same potentials $\Phi_{0}/2$ and $-\Phi_{0}/2$ as, $\Phi^{\alpha }(r_{0},0)=A(r_{0}^{2\alpha })=\Phi_{0}/2$ and $\Phi^{\alpha }(0,z_{0})=A(-2z_{0}^{2\alpha })=-\Phi_{0}/2$. With this conditions we can find $A=\frac{\Phi_{0}}{2r_{0}^{2\alpha }}$ and $A=\frac{\Phi_{0}}{4z_{0}^{2\alpha }}$; therefore, $r_{0}^{2\alpha }=2z_{0}^{2\alpha }$. Thereby, the fractional electrodes shape for the fractional potential in the presented Cantor–type cylindrical coordinates given by, $\Phi^{\alpha }(r,z)=\frac{\Phi_{0}}{2r_{0}^{2\alpha }}(r^{2\alpha }-2z^{2\alpha })=\pm 1$. The applied electric potential, $\Phi_{0}$ (that is applied to the hyperbolic rod’s) is either an r.f. potential $V_{0}\cos\Omega t$ or a combination of a d.c. potential, *U*, of the form,^1,13^ $\Phi_{0}=U-V_{0}\cos\Omega t$, where Ω=2πf is the angular frequency (in rad s^−1^) of the r.f. field, and *f* is the frequency in hertz. Using the given definitions and information, the fractional potential $\Phi^{\alpha }(r,z)$ can be defined as, $\Phi^{\alpha }(r,z)=\frac{1}{2r_{0}^{2\alpha }}(r^{2\alpha }-2z^{2\alpha })\left(U-V_{0}\cos\Omega t\right)$.

**Figure 2.**
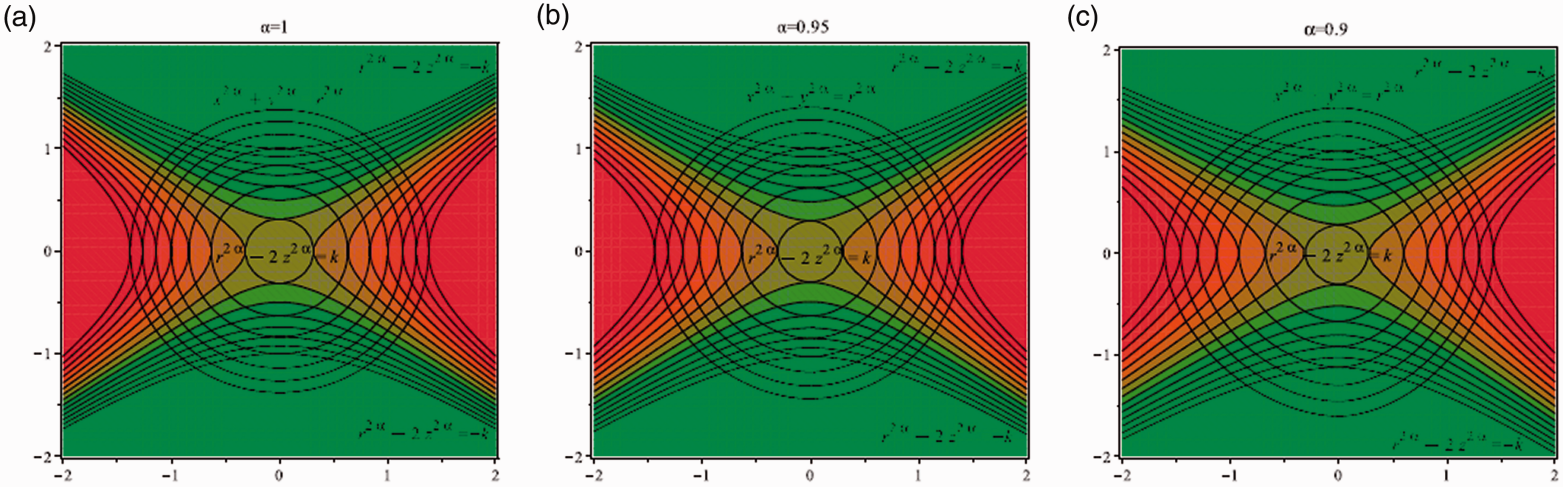
Field lines of electric fields; (a): *α* = 1, (b): α=0.95 and (c): α=0.9.

**Figure 3.**
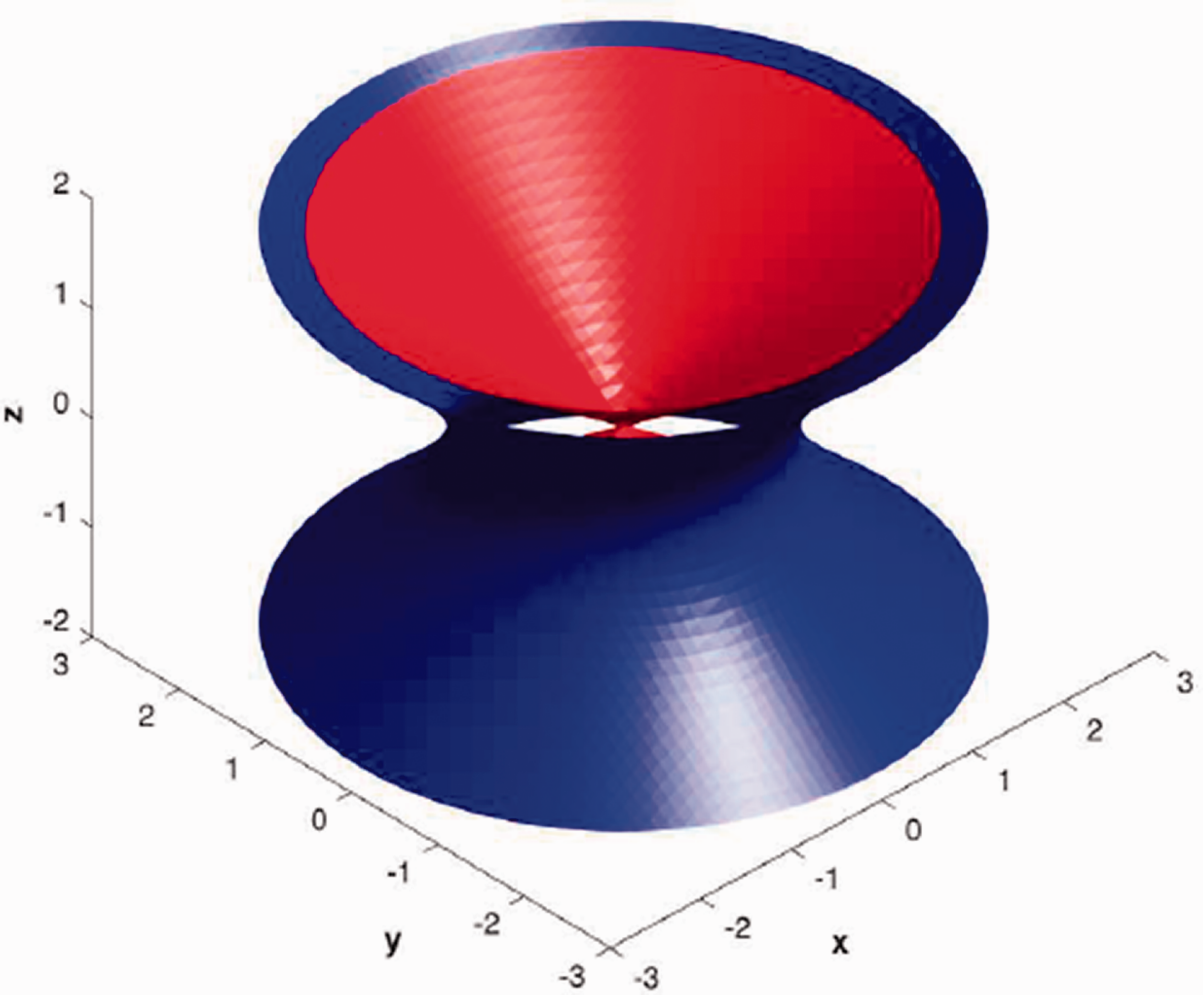
Ion trap simulation in 3D when *α* = 1, ring: $ax^{2}+by^{2}-cz^{2}=1$ and end cap: $ax^{2}+by^{2}-cz^{2}=-1$ with *a* = 1, *b* = 1, *c* = 2.

The map of the electric field inside the trap and 3D simulations for α=0.9,0.95,1 are shown in Figure 2(a) and (b), while 3D simulation for the classical Paul trap (*α* = 1) is shown in Figure 3.

**Figure 4.**
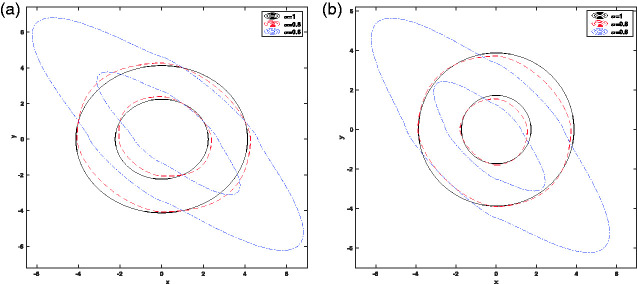
Contour lines when α=1,0.8,0.6 and *a* = 1, *b* = 1, *c* = 2; (a): ring: $ax^{2\alpha }+by^{2\alpha }-cz^{2\alpha }=1$ and (b): end cap: $ax^{2\alpha }+by^{2\alpha }-cz^{2\alpha }=-1$.

As can be seen in Figure 2(a) and (b), for *α* = 1, equation $x^{2}+y^{2}=r^{2}$ shows a circle, for α=0.95 equation $x^{1.9}+y^{1.9}=r^{1.9}$ represents a smaller irregular circle compared with *α* = 1 and for α=0.9, equation $x^{1.8}+y^{1.8}=r^{1.8}$ indicates a smaller irregular circle compared to α=0.95 and *α* = 1. In Figure 3, the ring and end cap equations are obtained from $ax^{2\alpha }+by^{2\alpha }-cz^{2\alpha }=1$ and $ax^{2\alpha }+by^{2\alpha }-cz^{2\alpha }=-1$, respectively. Figure 4(a) and (b) indicate the contour lines for ring: $ax^{2\alpha }+by^{2\alpha }-cz^{2\alpha }=1$ and end cap: $ax^{2\alpha }+by^{2\alpha }-cz^{2\alpha }=-1$, when α=1,0.8,0.6 and *a* = 1, *b* = 1, *c* = 2. According to these figures, it can be concluded that by reducing *α* from 1 to 0.6, the contour lines along the axis y=−x become more elongated.

## Fractional motion of trapped ions in the Paul-trap

In this section, the motion of ion inside a Paul–trap with the fractional potential in the Cantor-type cylindrical coordinates was modelled. The relationship between force, mass, and the applied fields in Newton’s second law and the Lorentz force law is as follows,

(13) F=ma (Newton's second law)   and   F=qE (Lorentz force law without magnetic field)

where, **F** is the force applied to the ion, **m** is the mass of the ion, **a** is the acceleration, **q** is the ionic charge and **E** is the electric field. Here, **F**, **a** and **E** are vectorial variables. The electric field components in the trap with the fractional potential are as follows,

(14) [Erα Ezα]=Eα=−∇αΦ(r,z)=[−∂α∂rαΦ(r,z) −∂α∂zαΦ(r,z)]=[−22αΓ(α+32)π(2α+1)r02α(U−Vcos⁡Ωt)rα 22α+1Γ(α+32)π(2α+1)r02α(U−Vcos⁡Ωt)zα]

Therefore, the equations of motion for the only positive ion in the Paul–trap with the fractional potential in the Cantor–type cylindrical coordinates without using the magnetic field are given by,

(15) $$\{\begin{array}{l} \frac{d^{2}z}{d\xi }+\left(a_{z}-2q_{z}\cos2\xi \right)\frac{2^{2\alpha }\Gamma (\alpha +\frac{3}{2})}{\sqrt{\pi }(2\alpha +1)}z^{\alpha }=0,\\ \frac{d^{2}r}{d\xi }+\left(a_{r}-2q_{r}\cos2\xi \right)\frac{2^{2\alpha }\Gamma (\alpha +\frac{3}{2})}{\sqrt{\pi }(2\alpha +1)}r^{\alpha }=0 \end{array}$$

with $r_{0}^{2\alpha }=2z_{0}^{2\alpha }$ and the assumptions,

(16) $$\xi =\frac{\Omega t}{2},\;a_{z}=-2a_{r}=-\frac{4qU}{mr_{0}^{2\alpha }\Omega^{2}},\;q_{z}=-2q_{r}=\frac{2qV}{mr_{0}^{2\alpha }\Omega^{2}}$$

Assuming *α* = 1, basic motion equations are as follows,

(17) $$\{\begin{array}{l} \frac{d^{2}z}{d\xi^{2}}+\left(a_{z}-2q_{z}\cos2\xi \right)z=0,\\ \frac{d^{2}r}{d\xi^{2}}+\left(a_{r}-2q_{r}\cos2\xi \right)r=0 \end{array}$$

## Programming and numerical simulations

In this section, programming and numerical simulations of the dynamical system for the trapped ion inside Paul–trap are investigated and discussed. For programming and numerical simulations, the charge state of +1 was considered. We first plot stability regions in the (*a*, *q*) and (V,−U) plans, ion trajectories in time, the evolution of phase space ion path, resolution of the ion trap and fractional resolution of the ion trap. Then, we study and discuss the effect of the fractional potential on the mass resolution. The effect of the fractional potential was examined for ions of ^131^Xe and ^132^Xe.

### Ion trajectories

**Figure 5.**
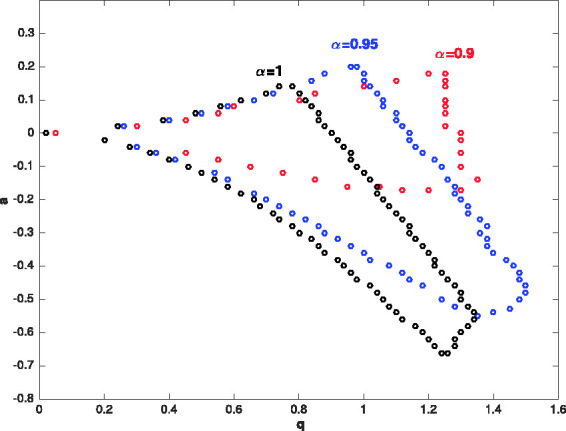
The first stability region of Paul–trap when α=1,0.95,0.9.

**Figure 6.**
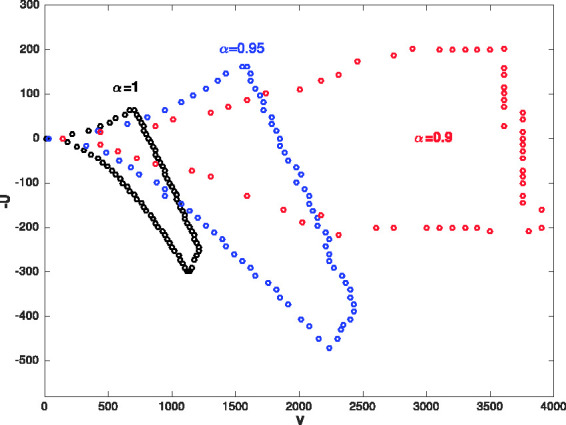
The stability diagram in (V,−U) plan for ^131^Xe with $\Omega =2\pi \times 1.05\times 10^{6}$ rad/s, $z_{0}=0.707$ mm and α=1,0.95,0.9.

**Figure 7.**
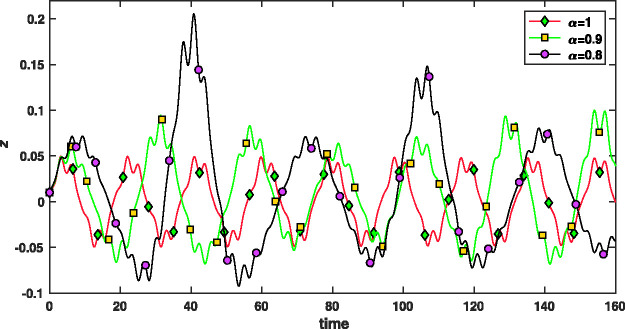
The ion trajectories in time for $q_{z}=0.9$ and α=0.8,0.9,1.

Figure 5 shows the first stability region of Paul–trap with the fractional potential when α=1,0.95,0.9. As can be seen, changing the fractional parameter *α* from 1 to 0.9, first stability region will be smaller along the *a* axis and bigger along the *q* axis. The stability diagrams (V,−U) plane for ^131^Xe with $\Omega =2\pi \times 1.05\times 10^{6}$ rad/s, $z_{0}=0.707$ mm and α=1,0.95,0.9 have been shown in Figure 6. When the fractional parameter *α* decreases from 1 to 0.9, the stability diagrams (V,−U) plane become larger. Figure 7 presents the ion trajectories in time when *a_z_* = 0, $q_{z}=0.9$ and α=0.8,0.9,1, respectively. This figure show that the ion trajectories are comparable for all values of the fractional parameters α=0.8,0.9 and 1. However, as the value of the parameter *α* decreases, the ion rotation space increases.

**Figure 8.**
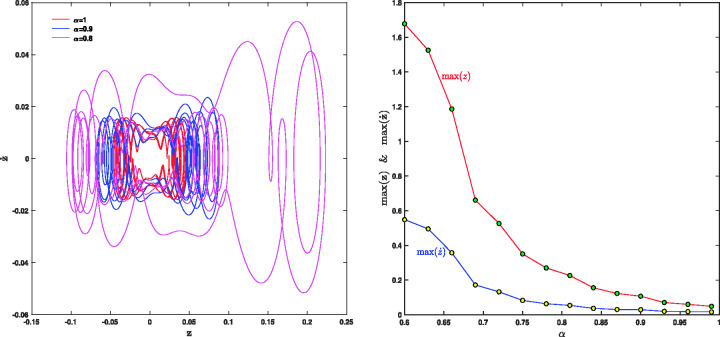
Left: ion trajectories in $z-\dot{z}$ plane for $q_{z}=0.32$ and α=0.8,0.9,1; Right: max⁡(z) and $\max(\dot{z})$ vs *α*.

The ion trajectories in $z-\dot{z}$ plane for $q_{z}=0.32$, α=0.8,0.9,1 are shown in Figure 8. The left side and right sides of this figure show the max⁡(z) and $\max(\dot{z})$ versus fractional parameter *α*. As can be seen in the right side, the rotation space of the ions increases as the value of *α* decreases from 1 to 0.8. As the left side shows, by decreasing the value of *α* from 1 to 0.6, the values of max⁡(z) and $\max(\dot{z})$ increase, but max⁡(z) is increasing faster than $\max(\dot{z})$. As Figure 8 shows, there are two periodic attractors in the system, which are corresponding to forced oscillations confined to the left or right well. The portraits of the phase obviously reflect the existence of one or two attractors and of fractal basin boundaries for the trapped ion, assimilated with a periodically forced double well oscillator. The system can converge rapidly to one of the two attractors, based on the initial conditions and fractional parameter *α*. Generally the attraction basins have a complicated shape, and the boundary between them is fractal.^24,46^

**Figure 9.**
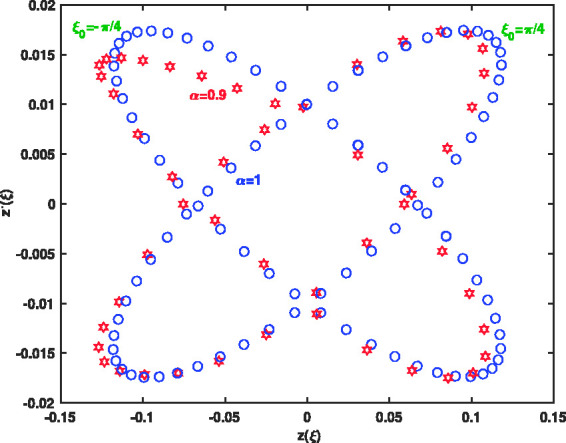
Evolution of phase space ion trajectory for different values of the phase $\xi_{0}=\frac{\pi }{4}$ and $\xi_{0}=-\frac{\pi }{4}$ when α=0.9 and *α* = 1.

Figure 9 shows the mechanical properties of the confined ions analyzed through the ion displacements in the phase space. Phase space ion trajectory for different values of r.f. fields with initial phases $\xi_{0}=\frac{\pi }{4}$ and $\xi_{0}=-\frac{\pi }{4}$ has been proposed for α=0.9 and *α* = 1. The computational results in this figure show the comparable phase space for different values of fractional parameter α=0.9 and *α* = 1.

### Effect of the fractional factor on the mass resolution

This section presents the effect of fractional parameter *α* on the mass resolution of trapped ions. As we know, the resolution of a Paul–trap mass spectrometer is a function of the mechanical accuracy of the hyperboloid of the ion trap, $\Delta r_{0}$ and the stability performances of the electronics device, such as variations in voltage amplitude ΔV and the r.f. frequency ΔΩ.^46^ The computational resolution will tell us how accurate the form of the voltage signal is. To derive a theoretical formula for the fractional resolution, according to equation (17), there will be,

(18) $$q_{z}=\frac{2eV}{mr_{0}^{2\alpha }\Omega^{2}}$$

Considering the partial derivatives on the variables of the stability parameters, expression of the resolution Δm can be computed as follows,

(19) $$\Delta m=2\alpha \left(\frac{2eV}{r_{0}^{2\alpha +1}\Omega^{2}q_{z}}\right)|\Delta r_{0}|+\left(\frac{2e}{r_{0}^{2\alpha }\Omega^{2}q_{z}}\right)|\Delta V|+2\left(\frac{2eV}{r_{0}^{2\alpha }\Omega^{3}q_{z}}\right)|\Delta \Omega |$$

then, there will be,

(20) $$\Delta m=2\alpha \left(\frac{2eV}{r_{0}^{2\alpha }\Omega^{2}q_{z}}\right)|\frac{\Delta r_{0}}{r_{0}}|+\left(\frac{2eV}{r_{0}^{2\alpha }\Omega^{2}q_{z}}\right)|\frac{\Delta V}{V}|+2\left(\frac{2eV}{r_{0}^{2\alpha }\Omega^{2}q_{z}}\right)|\frac{\Delta \Omega }{\Omega }|$$

therefore, we have,

(21) $$\Delta m=m\left(2\alpha |\frac{\Delta r_{0}}{r_{0}}|+2|\frac{\Delta \Omega }{\Omega }|+|\frac{\Delta V}{V}|\right)$$

Thus, the fractional resolution is given by,

(22) $$\frac{m}{\Delta m}={\left(2\alpha |\frac{\Delta r_{0}}{r_{0}}|+2|\frac{\Delta \Omega }{\Omega }|+|\frac{\Delta V}{V}|\right)}^{-1}$$

**Figure 10.**
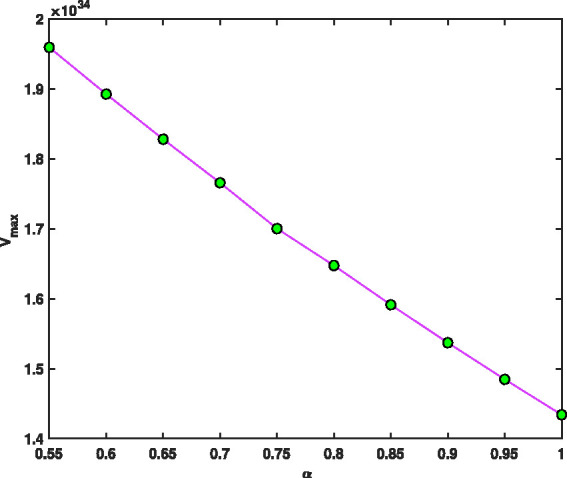
Maximum values of *V*, *V*_max_, as a function of fractional parameter *α*, when $\Omega =2\pi \times 1.05\times 10^{6}$ rad/s, $z_{0}=0.707$ mm and *a* = 0.

**Figure 11. fig11-14690667211026790:**
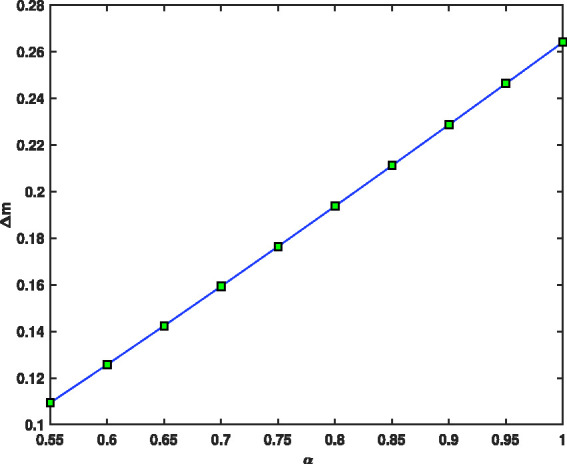
Spacing between two signals, Δm, as a function of fractional parameter *α*.

Uncertainties $|\frac{\Delta \Omega }{\Omega }|=10^{-7}$, $|\Delta V|=10^{-4}$ and $|\Delta r_{0}|=10^{-3}$ have been used for voltage, r.f. and geometry for fractional mass resolution,^46^ respectively. Assuming that $\Omega =2\pi \times 1.05\times 10^{6}$ rad/s, $z_{0}=0.707$, *a* = 0, maximum values of voltage *V*, *V*_max_, as a function of the fractional parameter, *α*, and function of ion mass, *m*, for ^131^Xe and 1^132^Xe when *m* = 131, 132 and α=0.9,1,^46^ are presented in Figure 10. As can be seen, by increasing the fractional parameter a from 0.55 to 1, the maximum voltage, *V*_max_, decreases rapidly like a negative exponential function. Figure 11 shows the spacing between two signals, Δm, as a function of the fractional parameter *α*. As can be seen, by increasing the fractional parameter *α* from 0.55 to 1, the spacing between two signals, Δm, decreases rapidly like an exponential function. This means that, by reducing the fraction parameter *α*, the separation can be performed more accurately.

**Figure 12.**
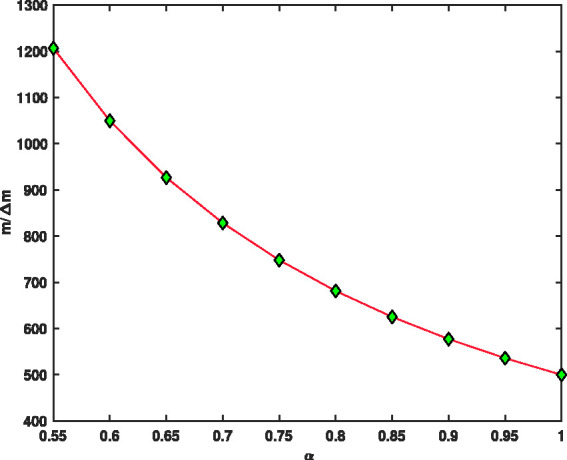
The fractional resolution of ion trap, m/Δm, as a function of fractional parameter *α*.

Figure 12, indicates the fractional mass resolution m/Δm as a function of fractional parameter *α*. The results of this figure show that by decreasing the fraction parameter *α* from 1 to 0.55, the fractional mass resolution values rapidly increase from 400 to 1200. The higher fractional mass resolution indicates better and more accurate separation. In Figures 10 [Fig fig11-14690667211026790]to 12, to find the vertical values, we divided the interval α=[0.55,1] into *N* = 45 parts using the stepsize *h* = 0.01, then *V*_max_, Δm and m/Δm values were found in all these points. Then, all the curves were plotted using the 45 found points, but to make the curves easily visible, the markers have been used only in ten points.

**Figure 13.**
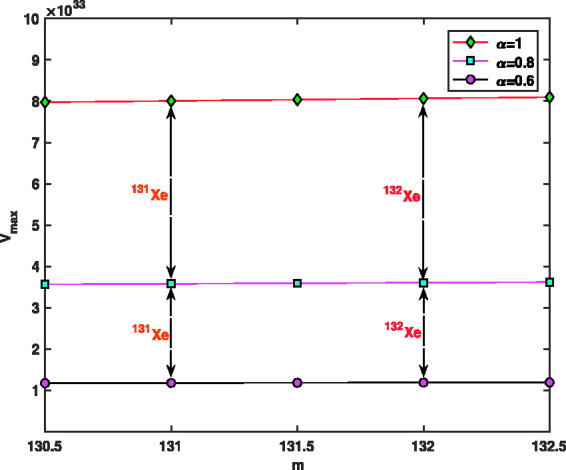
Maximum values of *V*, *V*_max_, as a function of ion mass, *m*, when $\Omega =2\pi \times 1.05\times 10^{6}$ rad/s, $z_{0}=0.707$ mm, *a* = 0 and α=0.6,0.8,1.

**Figure 14.**
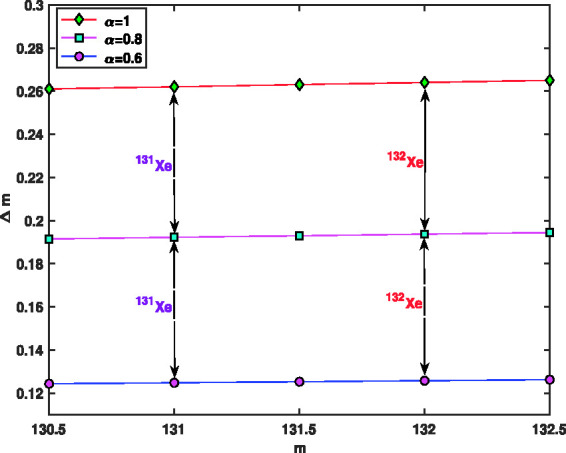
Spacing between two signals, Δm, as a function of ion mass, *m*, when α=0.6,0.8,1.

Maximum values of *V*, *V*_max_, as a function of ion mass, *m*, for hypothetical values $\Omega =2\pi \times 1.05\times 10^{6}$ rad/s, $z_{0}=0.707\;\mathrm{mm}$, *a* = 0 and α=0.6,0.8,1 is shown in Figure 13. This figure also shows the maximum values of voltage *V* for the ions ${\;}^{131}Xe$ and ${\;}^{132}Xe$ when the fractional values are α=0.6, α=0.8 and *α* = 1. As can be seen, the maximum voltage for α=0.6 and α=0.8 is less than the maximum voltage for *α* = 1; and lower voltage indicates better and more accurate separation. Figure 14 presents the spacing between two signals, Δm, as a function of ion mass, *m*, when the fractional parameters are α=0.6, α=0.8 and *α* = 1. This figure also shows the values of spacing between two signals for the ions ${\;}^{131}Xe$ and ${\;}^{132}Xe$. As can be seen, ${\left(\Delta m\right)}_{\alpha =0.6}<{\left(\Delta m\right)}_{\alpha =0.8}<{\left(\Delta m\right)}_{\alpha =1}$, and less Δm indicates better and more accurate separation. In Figures 13 and 14, to find the vertical values, we divided the interval m=[130.5,132.5] into *N* = 40 parts using the stepsize *h* = 0.05, then the values of *V*_max_ and Δm were found in all these points. All the curves were plotted using the 45 found values, but to make the curves easily visible, the markers have been used only in five points.

## Conclusion

A modified three-dimensional radio frequency Paul–trap with fractional potential was introduced in this study. The first stability region in (*q*, *a*) and (V,−U) planes was also shown. Moreover, effect of fractional parameter *α* on the mass separation was studied. Maximum values of voltage, *V*_max_, as a function of the fractional parameter *α* for was derived for ions ^131^Xe and ^132^Xe assuming that $\Omega =2\pi \times 1.05\times 10^{6}$ rad/s, $z_{0}=0.707$ mm and *a* = 0. Further, the spacing between two signals, Δm, and mass fractional resolution, m/Δm, for ions ^131^Xe and ^132^Xe as a function of the fractional parameter *α* was studied and discussed. The fractional resolution of ion traps m/Δm increases when the fractional parameter *α* decreases. As was observed, with decreasing the fractional parameter a from 1 to 0.55, the fractional mass resolution rapidly increased from 400 to 1200. The high fractional resolution in good separation has high mass accuracy. As shown, the maximum voltage for α=0.6 and α=0.8 was less than the maximum voltage for *α*; and lower voltage indicates better and more accurate separation. The general results of this paper showed that the fractional parameter *α* can be an important and effective controller to optimize ion mass separation.

## Supplemental Material

sj-pdf-1-ems-10.1177_14690667211026790 - Supplemental material for Theoretical fractional formulation of a three-dimensional radio frequency ion trap (Paul-trap) for optimum mass separation Click here for additional data file. Supplemental material, sj-pdf-1-ems-10.1177_14690667211026790 for Theoretical fractional formulation of a three-dimensional radio frequency ion trap (Paul-trap) for optimum mass separation by Sarkhosh Seddighi Chaharborj, Shahriar Seddighi Chaharborj, Zahra Seddighi Chaharborj and Pei See Phang in European Journal of Mass Spectrometry

sj-pdf-2-ems-10.1177_14690667211026790 - Supplemental material for Theoretical fractional formulation of a three-dimensional radio frequency ion trap (Paul-trap) for optimum mass separation Click here for additional data file. Supplemental material, sj-pdf-2-ems-10.1177_14690667211026790 for Theoretical fractional formulation of a three-dimensional radio frequency ion trap (Paul-trap) for optimum mass separation by Sarkhosh Seddighi Chaharborj, Shahriar Seddighi Chaharborj, Zahra Seddighi Chaharborj and Pei See Phang in European Journal of Mass Spectrometry
