# Supplementary material for: Theoretical fractional formulation of a three-dimensional radio frequency ion trap (Paul-trap) for optimum mass separation
Source: Eur J Mass Spectrom (Chichester). 2021 Jul 4;27(2-4):73–83. doi: 10.1177/14690667211026790 (PMC8422591; doi:10.1177/14690667211026790)
Supplement: sj-pdf-1-ems-10.1177_14690667211026790 - Supplemental material for Theoretical fractional formulation of a three-dimensional radio frequency ion trap (Paul-trap) for optimum mass separation [file sj-pdf-1-ems-10.1177_14690667211026790.pdf]

We warmly thank the very competent Editor and Reviewers for the time spent on reading the manuscript and for the valuable comments and suggestions. We have modified the manuscript accordingly, and the detailed corrections are listed below point by point.

Whole paper checked and edited according Reviewers comments.

Reviewer: 1

Comments to the Author
This is a review of the revised version R1 of the manuscript “Theoretical Fractional Formulation of a Three-dimensional Radio Frequency Ion Trap (Paul-Trap) for Optimum Mass Separation”.

Some of the reviewers’ comments have been addressed by the authors. However, there are still many issues open with this manuscript.

One of the main points, mentioned by both reviewers, is the problem with using correct English. Although a lot has been corrected, there are still too many mistakes in the text. This includes the old text written in black, but also the new text written in different colors. The first six sentences of the introduction already contain four cases of wrong wording and one typo. The whole text has to be re-checked, and I would also advise to seek help from native English speakers.

Another point is the fractional parameter alpha (note: The text jumps back and forth between using the word fraction or fractional; it should be fractional as far as I can see it): This parameter, as well as the fractional derivatives, is normally used in order to obtain a better representation of experimental data; better means here that the calculated values are closer to the corresponding experimental ones. Especially in irregular (“chaotic”) data, or data containing spikes, the fractional derivative can help to obtain a better mathematical formulation, yielding calculated values closer to the experimental values including e.g. spikes.
This paper goes in a different direction. The fractional parameter alpha is introduced as if it was available as another experimental parameter that can be used to tune the resolving power. The use of fractional derivatives brings the parameter alpha into the equations, and then the resolving power has been reformulated with this new parameter, and the dependence of the resolving power on alpha has been demonstrated. However, this alpha (i.e., its mathematical form in the equation for resolving power) depends on the definition of fractional derivatives, of which there are many (and not all of them yield the same values). Furthermore, how should this alpha be changed or controlled in an experimental set up? Alpha is an abstract parameter; it does not exist in experimental reality. Therefore, using it to tune the resolving power is for me not logic; if the authors see this differently, they need to proof and explain this; I would expect at least a whole page of text or even more to justify this approach of using abstract mathematical parameters as experimentally controlled parameters. Just showing the graphs with dependencies on alpha are not sufficient. Finally, the paper does not contain a single experimental example that fractional derivatives are helpful in quadrupol mass spectrometry, so the whole concept might not be useful here.

a. All “fraction” changed to “fractional”.
b. New section added to Introduction as follows,

History of Fractional Calculus

By looking at articles published in recent decades in the fields of science and engineering, we get acquainted with the topics of fractional calculus, differential equations with fractional derivatives, and concepts of this kind. So far, many books and papers in this field have been written from theoretical and practical points of view [25-31]. The subject of fractional calculus is more than 300 years old. The idea of fractional calculus dates back to the time of basic or classical calculus, and most theories about it were developed before the twentieth century. This was first introduced by Leibniz and L'Hospital's in 1653.
In the twentieth century, many efforts were made by various scientists in this field. Caputo, by rewriting Riemann-Liouville formula, introduced a new derivative that is now used under the name Caputo derivative. Notable people who have worked on this topic during this period are: Hardy, Samko, Weyl, Riesz and Blair. Since 1970 until now, many people have studied in this field and also left useful articles and books. In this regard, Spanier, Oldham, Miller, Kilbas, Ross and Podlubny can be mentioned. The best resources for studying fractional calculations are books and articles of Miller and Ross, Kilbas and Podlubny. See reference [32] for a more comprehensive study of the history of fractional calculus.

c. Explanation about fractional parameter:

All comments carefully edited and revised.

According the relations in the equation (3) we have,

$$a_z \propto \frac{1}{r_0^{2\alpha}}, \quad q_z \propto \frac{1}{r_0^{2\alpha}}, \quad U \propto r_0^{2\alpha}, \;\; V \propto r_0^{2\alpha},$$

therefore, with decreasing the fractional parameter α from 1 to 0, values of a_z and q_z decreasing and values of U and V increasing. For $z_0 = 0.707$ mm and $\alpha = 1, 0.9$, we have,

$$r_0^{2\alpha} = 2z_0^{2\alpha} \quad \Longrightarrow \quad r_0^{2\alpha} = 2(0.707)^{2\alpha},$$

for $\alpha = 1 \quad \Longrightarrow \quad \begin{cases} a_z \propto \frac{1}{2(0.707)^2} = 1 \\ q_z \propto \frac{1}{2(0.707)^2} = 1 \\ U \propto 2(0.707)^2 = 0.99 \\ V \propto 2(0.707)^2 = 0.99 \end{cases}$

for $\alpha = 0.9 \quad \Longrightarrow \quad \begin{cases} a_z \propto \frac{1}{2(0.707)^2} = 0.93 \\ q_z \propto \frac{1}{2(0.707)^2} = 0.93 \\ U \propto 2(0.707)^2 = 1.07 \\ V \propto 2(0.707)^2 = 1.07 \end{cases}$

in this case fractional parameter α can act as controller and can control stability regions in the $a_z - q_z$ plane and $U - V$ plane. And according the equations (6) and (7), spacing between two signals, Δm, and fractional resolution, $\frac{m}{\Delta m}$, we have,

$$\Delta m \propto \Psi(\alpha, V), \qquad and \qquad \frac{m}{\Delta m} \propto \Psi^{-1}(\alpha, V),$$

therefore, by controlling the fractional parameter α can control Δm and $\frac{m}{\Delta m}$.

Presented figures, show that with controlling the parameter α can control those and may can find optimize α for any special ion.

Further points:
There are still theorems in the paper without proofs. If you like to continue to use the word "theorem", please write at least that you omit the proofs, and give references where to find them. Theorems without proofs are highly unusual.

Proof of Theorems added.

"beta=alpha": As far as I see it, beta is still chosen to be the same as alpha, only all the text mentioning this choice has been deleted. Deleting text is not a justification; so there still needs to be an explanation why beta has been chosen that way (and can be chosen that way), Theorem 2.3 clearly states the presence of beta.

Edited and explained as referees comment.

According Theorem 1.2 we have,

$$D^{\alpha}\xi^{\beta} = \frac{\Gamma(\beta+1)}{\Gamma(\beta-\alpha+1)}\xi^{\beta-\alpha}, \quad \xrightarrow{\alpha \to 2\alpha} \quad D^{2\alpha}\xi^{\beta} = \frac{\Gamma(\beta+1)}{\Gamma(\beta-2\alpha+1)}\xi^{\beta-2\alpha}; \quad \frac{n-1}{2} < \alpha \leq \frac{n}{2},$$
$$\xrightarrow{\beta \to 2\beta} \quad D^{2\alpha}\xi^{2\beta} = \frac{\Gamma(2\beta+1)}{\Gamma(2\beta-2\alpha+1)}\xi^{2\beta-2\alpha}; \quad 2\beta > 0\,.$$

for $\xi^{2\beta} = x^{2\alpha}$,

$$D^{2\alpha}x^{2\alpha} = \frac{\Gamma(2\alpha+1)}{\Gamma(2\alpha-2\alpha+1)}x^{2\alpha-2\alpha} = \frac{\Gamma(2\alpha+1)}{\Gamma(1)}x^{0}.$$

....

Similarly, the choice of gamma1, gamma2 and gamma3. Please write explicitly that this choice is necessary to obtain the trapping potential of the desired form.
Has been corrected as,

From equation (3) it results that $\gamma_1 + \gamma_2 + \gamma_3 = 0$, when $\nabla^{2\alpha}\phi^{\alpha} = 0$. For an ion trap, $\gamma_1 = \gamma_2 = 1$ and $\gamma_3 = -2$ and for a quadrupole mass filter $\gamma_1 = -\gamma_2 = 1$ and $\gamma_3 = 0$. In this paper, we focused on the Paul-ion trap, then we used, $\gamma_1 = \gamma_2 = 1$ and $\gamma_3 = -2$.

Figure 9-11. real time: It is still not clear what real time should mean here, these are simulation/calculation results. What is the specific meaning of "real" in this context?
All "real time" corrected to "time" in the draft paper.

in this draft "real time" means, time in any unit of time in the laboratory.

Figures 8-17: These are dependencies of different quantities on alpha. But alpha is only an abstract parameter without an experimental representation. What are these graphs then useful for? Please see also the introductory comment on alpha. If the authors cannot justify and explain how alpha can be used in experiments, the whole discussion based on the graphs is purely theoretic and not helpful. I would like the authors to really be specific if they disagree.

According the relations in the equation (3) we have,

$$a_z \propto \frac{1}{r_0^{2\alpha}}, \quad q_z \propto \frac{1}{r_0^{2\alpha}}, \quad U \propto r_0^{2\alpha}, \ \ V \propto r_0^{2\alpha},$$

therefore, with decreasing the fractional parameter α from 1 to 0, values of a_z and q_z decreasing and values of U and V increasing. For $z_0 = 0.707$ mm and $\alpha = 1, 0.9$, we have,

$$r_0^{2\alpha} = 2z_0^{2\alpha} \quad \Longrightarrow \quad r_0^{2\alpha} = 2(0.707)^{2\alpha},$$

$$\text{for } \alpha = 1 \quad \Longrightarrow \quad \begin{cases} a_z \propto \frac{1}{2(0.707)^2} = 1 \\ q_z \propto \frac{1}{2(0.707)^2} = 1 \\ U \propto 2(0.707)^2 = 0.99 \\ V \propto 2(0.707)^2 = 0.99 \end{cases}$$

$$\text{for } \alpha = 0.9 \quad \Longrightarrow \quad \begin{cases} a_z \propto \frac{1}{2(0.707)^2} = 0.93 \\ q_z \propto \frac{1}{2(0.707)^2} = 0.93 \\ U \propto 2(0.707)^2 = 1.07 \\ V \propto 2(0.707)^2 = 1.07 \end{cases}$$

in this case fractional parameter α can act as controller and can control stability regions in the $a_z - q_z$ plane and $U - V$ plane. And according the equations (6) and (7), spacing between two signals, Δm, and fractional resolution, $\frac{m}{\Delta m}$, we have,

$$\Delta m \propto \Psi(\alpha, V), \qquad and \qquad \frac{m}{\Delta m} \propto \Psi^{-1}(\alpha, V),$$

therefore, by controlling the fractional parameter α can control Δm and $\frac{m}{\Delta m}$.

Presented figures, show that with controlling the parameter α can control those and may can find optimize α for any special ion.

Figures 8 up to 17 are following these results.
