# Supplementary material for: Theoretical fractional formulation of a three-dimensional radio frequency ion trap (Paul-trap) for optimum mass separation
Source: Eur J Mass Spectrom (Chichester). 2021 Jul 4;27(2-4):73–83. doi: 10.1177/14690667211026790 (PMC8422591; doi:10.1177/14690667211026790)
Supplement: sj-pdf-2-ems-10.1177_14690667211026790 - Supplemental material for Theoretical fractional formulation of a three-dimensional radio frequency ion trap (Paul-trap) for optimum mass separation [file sj-pdf-2-ems-10.1177_14690667211026790.pdf]

We warmly thank the very competent Editor and Reviewers for the time spent on reading the manuscript and for the valuable comments and suggestions. We have modified the manuscript accordingly, and the detailed corrections are listed below point by point.

Whole paper checked and edited according Reviewers comments.

Reviewer: 2

Comments to the Author

1. In general, you have corrected a lot of flaws in your new manuscript. Your article has become way better from a scientific point of view with all the new additions. Unfortunately, there is still a lot of wrong use of grammar and/or wording. Nearly every sentence is missing a verb or is simply unfinished. Without a meticulous checking and correction of the whole draft this article cannot be accepted.

Paper checked for gramper and English problems.

2. Further questions that occurred during checking your article are: Still, what is the point of showing figure 5 to 7? Since there is no difference to see by the naked eye it is pointless to show more than one.
   Corrected.

3. Why neutral Xenon particles for the simulations when you do all your introduction for positively charged ions? Later in the text you call them ions without indicating any charge.

No any serious reason to use “neutral Xenon”, just because in few of our previous published papers we used those ions for simulations results [1, 2, 3].

[1] Kiai, S. S., Chaharborj, S. S., Bakar, M. A., Fudziah, I. (2011). Effect of damping force on CIT and QIT ion traps supplied with a periodic impulse voltage form. Journal of Analytical Atomic Spectrometry, 26(11), 2247-2256.

[2] Chaharborj, S. S., Kiai, S. S., Bakar, M. A., Ziaeian, I., Fudziah, I. (2012). A new impulsional potential for a Paul ion trap. International Journal of Mass Spectrometry, 309, 63-69.

[3] Chaharborj, S. S., Kiai, S. M. S., Fudziah, I., Majid, Z. A. (2012). Study of quadrupole mass filter supplied with a new impulsional potential. European Journal of Mass Spectrometry, 18(5), 431-438.

In the introduction, has been added “neutral” and edited introduction.

Page 11 line 20: value increasing from 400 to 400?! Which one is wrong?

Corrected as:

the fractional mass resolution values increasing rapidly from 400 to 1200.
